# Addicted? Reduced host resistance in populations with defensive symbionts

**DOI:** 10.1098/rspb.2016.0778

**Published:** 2016-06-29

**Authors:** Julien Martinez, Rodrigo Cogni, Chuan Cao, Sophie Smith, Christopher J. R. Illingworth, Francis M. Jiggins

**Affiliations:** 1 Department of Genetics, University of Cambridge, Cambridge, UK; 2 Departamento de Ecologia, Instituto de Biociências, Universidade de São Paulo, 05508 900 São Paulo, SP, Brazil

**Keywords:** *Wolbachia*, *Drosophila melanogaster*, antiviral resistance

## Abstract

Heritable symbionts that protect their hosts from pathogens have been described in a wide range of insect species. By reducing the incidence or severity of infection, these symbionts have the potential to reduce the strength of selection on genes in the insect genome that increase resistance. Therefore, the presence of such symbionts may slow down the evolution of resistance. Here we investigated this idea by exposing *Drosophila melanogaster* populations to infection with the pathogenic *Drosophila* C virus (DCV) in the presence or absence of *Wolbachia*, a heritable symbiont of arthropods that confers protection against viruses. After nine generations of selection, we found that resistance to DCV had increased in all populations. However, in the presence of *Wolbachia* the resistant allele of *pastrel*—a gene that has a major effect on resistance to DCV—was at a lower frequency than in the symbiont-free populations. This finding suggests that defensive symbionts have the potential to hamper the evolution of insect resistance genes, potentially leading to a state of evolutionary addiction where the genetically susceptible insect host mostly relies on its symbiont to fight pathogens.

## 1. Introduction

Pathogens impose strong selection on populations leading to the evolution of numerous adaptations to resist attack, as exemplified by the diversity of immune defences. In addition to resistance mechanisms encoded by the nuclear genome, organisms can also be associated with symbionts that protect them against infection. These defensive symbionts have been found in a diverse array of taxa [1–6]. Many of the best-studied examples are vertically transmitted bacterial symbionts in arthropods, such as *Hamiltonella defensa* that protects the pea aphid against hymenopteran parasitoids [7] or *Wolbachia* that protects *Drosophila* and mosquitoes against viruses [4,8,9].

The evolution of resistance through symbionts likely differs from the evolution of resistance provided by host genes in several important ways. Although rare on an ecological timescale, over evolutionary times these host–symbiont associations are characterized by extensive horizontal transmission, with frequent gains and losses of the symbiont [10,11]. The acquisition of a defensive symbiont may be a fast way to immediately gain complex adaptations encoded by many genes [10]. This allows the horizontal transfer of adaptations between species, in an analogous way to plasmid transfers in bacteria [12]. On the other hand, these defensive symbionts can be a very costly form of defence [13–17]. For example, the *Wolbachia* strains that provide the strongest antiviral protection are associated with substantial reductions in other fitness-related traits, such as fecundity, male fertility, egg viability or lifespan [15,18,19]. This trade-off between protection and cost is thought to be mediated by *Wolbachia* density, as strong antiviral protection requires a high symbiont density [19–21].

The presence of a defensive symbiont may affect the evolution of resistance mechanisms encoded by the nuclear genome. The acquisition of a defensive symbiont can lead to a redundancy of function where both host and symbiont genes contribute to the same biological process. Therefore, the presence of a symbiont may reshape the fitness landscape of host nuclear genes by changing the strength of selection on these genes. This may be especially important because pathogens are continually evolving to evade or suppress host defences. Therefore, hosts may become more susceptible over time unless they are also evolving novel forms of defence. Potentially, a defensive symbiont could slow down the evolution of host-encoded defences. Indeed, by relaxing the selection on host genes, the presence of the symbiont may prevent the spread of new resistance alleles, resulting in a population composed of hosts genetically susceptible to pathogens. In an analogous example, resistance to parasitoid wasps was lost in *Drosophila sechellia*, likely as the result of this species feeding on fruit that contain a toxin that kills the parasitoids [22]. A similar loss of host-gene originated defences in host–symbiont associations would potentially leave the host population with an evolutionary addiction to its symbiont, as symbiont-free individuals would be vulnerable to infection.

The dynamics of host resistance genes, defensive symbionts and pathogens may be complex, as changes in the frequency of any one of these players may alter the frequency of the others. For example, the spread of a protective symbiont may reduce the prevalence of the pathogen, leading to negative frequency-dependent selection [3,23]. However, this will not always be the case. For example, *Wolbachia* bacteria commonly cause a reproductive manipulation called cytoplasmic incompatibility [24], and this could result in them being fixed within populations regardless of whether viruses are present. Similarly, some pathogens may have broad host ranges and be frequently transmitted between different species. In this case, the presence of a protective symbiont in a host species may have little effect on the rate at which this host is exposed to the pathogen if the dynamics of the pathogen is mostly influenced by its epidemiology in other species.

We have investigated these processes using the common insect symbiont *Wolbachia*. Many strains of *Wolbachia* can protect insects against viral infection, both increasing survival and reducing viral titres [4,8,20,21]. In most natural populations, *Drosophila melanogaster* is infected with a strain of *Wolbachia* that protects it against a wide range of RNA viruses, including a naturally occurring and highly pathogenic virus called *Drosophila* C virus (DCV) [25,26]. There is also considerable genetic variation in susceptibility to DCV that is caused by the insect genome, and 47% of this genetic variance can be explained by a single amino acid polymorphism in a gene called *pastrel* [27]*.* This was confirmed when flies from a different population were artificially selected for DCV resistance, which caused the resistant allele of *pastrel* to increase in frequency [28]. A number of other genes affecting DCV resistance have also been mapped [27,28] (C. Cao 2015, unpublished data), but these have always been of relatively small effect and the same gene has never been found by different studies.

Here we tested whether the defensive symbiont *Wolbachia* can slow down the rate at which insects evolve resistance to viruses. We exposed populations of *D. melanogaster* to DCV in the presence or absence of a protective *Wolbachia* strain for nine generations. We then measured DCV resistance in our populations after selection and followed changes in the frequency of the *pastrel* resistant allele. Our findings suggest that *Wolbachia* has the potential to slow down the evolution of host resistance.

## 2. Material and methods

### (a) Fly population and *Wolbachia* infection

We used an outbred population of *D. melanogaster* that was founded from 1526 isofemale lines collected in 2014 in Coventry (UK) using traps baited with bananas. This original population was kept in the laboratory in large numbers for five generations at 25°C on a standard cornmeal diet (1% agar, 8.75% dextrose, 7.5% maize, 2% yeast, 3% nipagin). In order to control for *Wolbachia* infection, we introgressed the nuclear background of the outbred population into a cytotype infected with the *Wolbachia* strain *w*MelCS. For this, 100 males of the outbred population were crossed to 100 females of the *w*MelCS_b DrosDel *w*^1118^ isogenic line described elsewhere [15,19]. This backcross was repeated for six generations (assuming no selection this would lead to an average of 98% of the nuclear genome being replaced). Three independent introgression replicates were performed (1WC, 2WC and 3WC, table 1). *Wolbachia*-cured counterparts of these introgressed populations (1TC, 2TC and 3TC, table 1) were generated by raising them on Ready Mix Dried Food (Philip Harris) supplemented with 0.03% w/v tetracycline for two generations. After introgression and tetracycline treatment, the *Wolbachia* infection status was checked by PCR on 30 females per population (electronic supplementary material, S1). In order to homogenize the gut microbiota between *Wolbachia*-infected populations and their uninfected counterparts, the tetracycline-treated populations were then raised for one generation on standard cornmeal food on which 50 males of the *Wolbachia*-infected populations had been kept for 1 day and removed. Experiments were all performed more than two generations after tetracycline treatment.

**Table 1.** Populations of *D. melanogaster* used in the selection experiment.

replicate population	*Wolbachia* infection status	selection treatment
1WC	*w*MelCS	control
2WC	*w*MelCS	control
3WC	*w*MelCS	control
1TC	no *Wolbachia*	control
2TC	no *Wolbachia*	control
3TC	no *Wolbachia*	control
1WDCV	*w*MelCS	DCV
2WDCV	*w*MelCS	DCV
3WDCV	*w*MelCS	DCV
1TDCV	no *Wolbachia*	DCV
2TDCV	no *Wolbachia*	DCV
3TDCV	no *Wolbachia*	DCV

### (b) Virus production and infection

The DCV was produced in Schneider *Drosophila* line 2 (DL2) cells as described in [29] (see protocol in electronic supplementary material, S1). To infect flies with DCV, 3–6 day old flies were anaesthetized with CO_2_, then were stabbed in the left pleural suture on the thorax with a 0.15 mm diameter anodized steel needle (Austerlitz Insect Pins) bent 0.25 mm from the end and dipped into viral solution. The DCV solution was prepared on the day of infection by defrosting an aliquot and diluting it in Ringer's solution [30] to a viral dose of 7.7 × 10^7^ TCID50 ml^−1^. Following infection flies were placed at 18°C.

### (c) Effect of *Drosophila* C virus infection with and without the symbiont

Within the gene *pastrel* the variant that is most strongly associated with resistance is a non-synonymous single nucleotide polymorphism (SNP) at position 521 (exon 6) that replaces Ala with Thr (named C521T [27]). We, therefore, used this SNP as a marker for the resistant allele of *pastrel* and measured the effect of DCV infection on the frequency of this SNP in our *Wolbachia*-free and *Wolbachia*-infected populations*.* We compared three treatments (100 female flies in each): no stabbing, stabbing with Ringer's solution and stabbing with DCV solution (see the infection procedure described earlier). Flies were placed at 18°C in a vial (20 females per vial) of standard cornmeal food and transferred to fresh vials every 3 days. Dead flies were counted every day for 15 days. At the end, the flies that survived were frozen for DNA extraction and genotyping (see methods in electronic supplementary material, S1).

### (d) Selection for virus resistance with and without the symbiont

Four different treatments were performed in parallel for nine generations with three replicate populations in each treatment: absence or presence of *Wolbachia*, infection with DCV or no viral infection (table 1). Populations were kept at 18°C in cages (90 mm diameter × 200 mm height) containing a 90 mm Petri dish of standard cornmeal food replaced every 3 days. For the DCV treatment, male and female flies were stabbed with DCV (as described above) at each generation. The experiment above showed that DCV infection led to strong selection favouring the resistant allele of *pastrel*, but not stabbing with DCV-free Ringer's solution (see Results). Therefore, no stabbing was performed for the control populations during selection. Our finding that *pastrel* confers resistance to DCV and not wounding is supported by previous work. It was shown that *pastrel* is specifically associated with increased survival after stabbing with DCV infection [27], and did not increase survival after flies were stabbed with other viruses. Similarly, no differences were found in DCV resistance over 34 generations of experimental evolution between populations that were stabbed with sterile medium and non-stabbed populations [28].

For each population, a given generation was started with 150 males and 150 females 3–6 day old flies placed in a cage. Given the high DCV-induced mortality in the *Wolbachia*-cured populations, two cages were prepared in order to obtain a sufficient number of offspring, leading to a population size of 300 males and 300 females for these populations (1TDCV, 2TDCV and 3TDCV, table 1). After 13 days, adult flies were discarded and the eggs kept for the next generation. At the start of the selection (generation 0), the DCV-induced mortality 13 days post-infection (dpi) was 50% and 20% for *Wolbachia*-cured and *Wolbachia*-infected populations, respectively. Eggs were collected from the Petri dish (changed on day 12) by pouring PBS solution (Thermo Fisher Scientific) onto the food and softly detaching the eggs from the food with a brush. For the *Wolbachia*-cured flies of the DCV treatment, eggs originating from the two cages of a same replicate population were pooled to ensure outcrossing. Using a pipette, 30 µl of the egg suspension (approx. 160 eggs) was transferred into a bottle of standard cornmeal food. Three bottles per replicate population were prepared, with the exception of the DCV-selected *Wolbachia*-cured populations for which six bottles were made. Bottles were placed at 25°C for larval development and adult emergence until the start of the next generation. Newly emerged flies that were not transferred to the cages were frozen at −20°C for later DNA extraction and genotyping (electronic supplementary material, S1).

At the end of the selection experiment, the within-host *Wolbachia* density of the *Wolbachia*-infected populations was quantified by quantitative PCR on DNA extracted from 10 pools of 10 females per population (electronic supplementary material, S1).

### (e) *Drosophila* C virus resistance assay

The level of resistance to virus infection was measured five generations after the selection experiment (see protocol above). Dead flies were recorded every day for 39 days after infection. For each infection treatment (DCV or Ringer control) and each replicate population, five independent vials were performed (100 flies in total). The same phenotypic assay was performed in parallel on the same populations but that were treated with tetracycline (for two generations, see protocol above) at the end of the selection experiment. Sixteen females per population from the same cohorts were genotyped and their *Wolbachia* infection status checked (electronic supplementary material, S1).

### (f) Selection and dominance coefficient estimates

An inference model was applied in order to estimate selection and dominance coefficients from the data. We first derive an expression for the relative fitness of the C allele at locus 521. We describe the fitness of the heterozygote and homozygote genotypes as

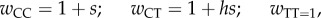
where *s* and *h* are, respectively, the selection and dominance coefficients. Assuming random mating, and that the C allele exists in the population with frequency *p* at some generation, the mean fitness of an individual genotype containing the C allele is given by

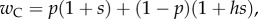
while the mean fitness of an individual genotype containing the T allele is

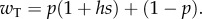


The ratio between these values is then given by

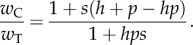


Expressing this in an alternative way, we then note that, if the mean fitness of an individual containing the T allele is rescaled to equal 1, the mean fitness of an individual containing the C allele may be expressed as 1 + *S*, where

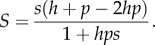


Using this result, we described the propagation of the system in terms of *p* using the delta method described in [31]. Where the mean and variance of *p* are given at generation *t* by *μ_t_* and 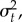 then ignoring mutation, the values of the equivalent parameters at generation *t* *+* 1 are approximated by

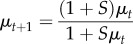


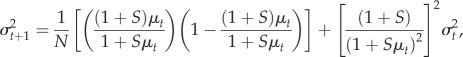
where *N* is the population size.

Observed values of the different genotype frequencies at times *t* were denoted as

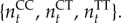


Setting σ^0^_2_ = 0 the parameters *μ_0_*, *s* and *h* were optimized in order to fit the genotype frequency observations. A multinomial model was used for fitting, integrating over the distribution of values of the frequency *p*. Parameters were thus optimized to maximize the likelihood

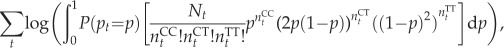
where *N_t_* was the total number of observations collected at time *t*, and

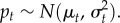


The integral was calculated via numerical approximation. Selection parameters were jointly inferred across replicate lines with or without *Wolbachia*; initial allele frequencies were learnt independently for each experimental replicate. Given maximum-likelihood estimates of *μ_0_*, *s* and *h*, the frequency *p_t_*, of the C allele at time *t* is normally distributed with mean *μ_t_* and variance 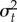; corresponding diploid allele frequencies may be estimated as *p_t_^2^*, *2p_t_*(1 − *p_t_*) and (1 − *p_t_*)*^2^*.

In order to measure uncertainty in the inferred parameters, repeated sampling of the evolutionary models for lines with and without *Wolbachia* was conducted, generating likelihood surfaces for the distributions of *s* and *h* in each circumstance*.* In order to evaluate the extent to which each evolved population was adapted to an environment without *Wolbachia*, approximate estimates of the final fitness of each population, under these conditions were calculated, being expressed relative to the final fitness of the line 1TDCV. Via repeated sampling, and considering the data without *Wolbachia*, we obtained sets of values {*s*_(*i*)_*, h*_(*i*)_*, μ*_9(*l,i*)_, *L*_(*i*)_} where *s*_(*i*)_ and *h*_(*i*)_ are proposed selection parameters, *μ*_9(*l*,*i*)_ are optimal mean allele frequencies at time *t* = 9 in each of three lines *l*, conditional on *s*_(*i*)_ and *h*_(*i*)_, and *L*_(*i*)_ are the associated log likelihoods. Given these values, we can calculate the approximate fitness values

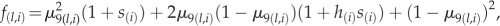
which can be expressed relative to those values obtained from the line 1TDCV as

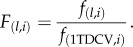


Denoting the value of *i* corresponding to the maximum-likelihood value *L*_(*i*)_ as i*, and the log-likelihood difference Δ_(i,i*)_ = *L*_(i*)_ − *L*_(*i*)_, then for lines grown without *Wolbachia*, relative fitness likelihood surfaces were calculated as the range [min*_i_ F*_(*l,i*)_, max*_i_ F*_(*l,i*)_] across the set of points *i* for which Δ_(i,i*)_ *≥* *ɛ* for variable log-likelihood difference cut-offs *ɛ*. To perform an equivalent calculation for lines grown with *Wolbachia*, multiple sets of selection parameters *s*_(*i*)_ and *h*_(*i*)_ were sampled from the no-*Wolbachia* data, along with log-likelihood differences Δ_(i,i*)_. Final mean frequencies *μ*_9(*l,j*)_ were then sampled from the with-*Wolbachia* data, along with their corresponding differences Δ_(l,j*)_, where j* denotes the optimal parameter set derived from the without *Wolbachia* data. Next, where

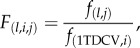
relative fitness likelihood surfaces were calculated as the range [min*_i,j_ F*_(*l,i,j*)_, max*_i,j_ F*_(*l,i,j*)_] across the set of points *i, j* for which Δ_(i,i*)_ *+* Δ_(j,j*)_ *≥* *ɛ* for variable log-likelihood difference cut-offs *ɛ*.

### (g) Statistical analyses

Statistical analyses were performed in the R software package [32]. Survival rates after DCV infection were analysed using Cox's proportional hazard mixed models (package coxme). The hazard ratio for a given replicate population is the probability of death occurring at a given timepoint divided by the probability of death in the control population. Flies that were alive at the end of the experiment were treated as censored data. Following the tests of the fixed effects, pairwise comparisons between selection treatments were performed with Tukey honest significance tests (Tukey HSD) using the package multcomp. The changes in allele frequency during the selection experiment were tested separately for the selected and control populations using a generalized linear model (package lme4) with a binomial distribution. *Wolbachia* densities were analysed using a linear mixed-effect model (package lme4), with the data being log-transformed to reach the assumptions of normality and homoscedasticity.

In all analyses, the selection treatment and the *Wolbachia* infection status were treated as fixed effects and the replicate population and/or vial of flies as random effects.

## 3. Results

### (a) The benefit conferred by host resistant allele depends on the symbiont infection status

**Figure 1.**
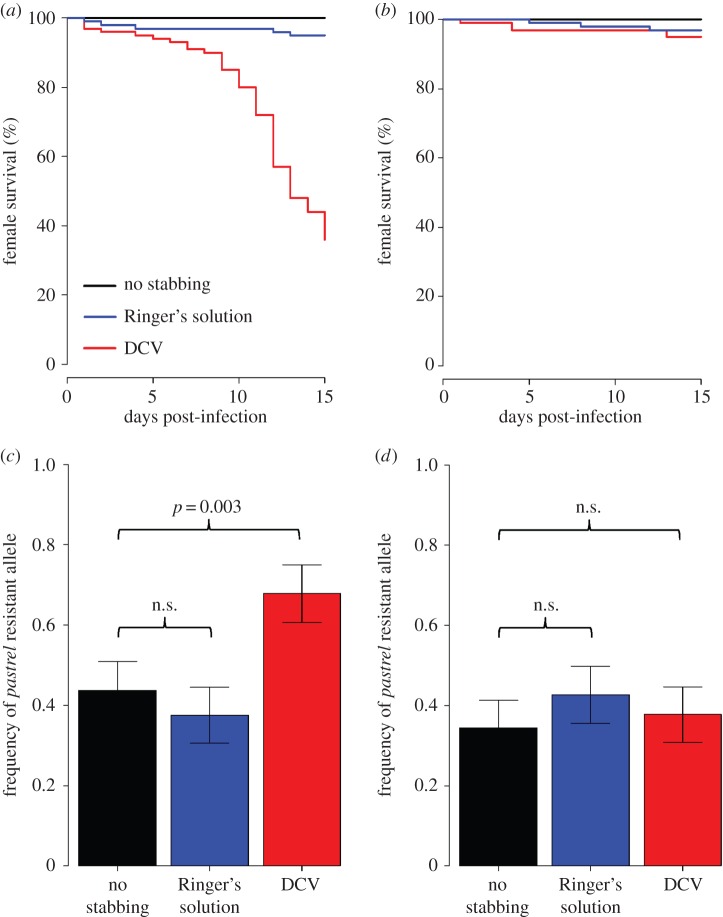
Effect of DCV infection on fly survival and the frequency of the resistant allele of *pastrel*. Survival of female flies following infection in (*a*) the *Wolbachia*-free and (*b*) the *Wolbachia*-infected populations. Frequency of the *pastrel* resistant allele in surviving flies 15 days after infection in (*c*) the *Wolbachia*-free and (*d*) the *Wolbachia*-infected populations. *p*-Values were obtained from a Dunnett's test comparing all treatments to the non-stabbed control flies. n.s., non-significant differences. Error bars are standard errors.

In natural *D. melanogaster* populations, most genetic variation in DCV resistance is caused by a polymorphism in a gene called *pastrel* [27]*.* We, therefore, assessed the effect of DCV infection on the survival of flies bearing the resistant and susceptible alleles of *pastrel* in our *Wolbachia*-free and *Wolbachia*-infected populations. Over 15 days post-infection we observed no mortality in non-stabbed flies, whereas stabbing with Ringer's solution induced around 5% mortality in both *Wolbachia*-free and *Wolbachia*-infected flies (figure 1*a,b*). The frequency of the *pastrel* resistant allele in the flies that survived was not significantly different between the Ringer and the ‘no stabbing’ treatments (figure 1*c,d*), indicating that the stabbing procedure does not select for or against the *pastrel* resistant allele.

By contrast, more than 60% of the flies stabbed with DCV died over 15 days in the absence of *Wolbachia*, whereas with *Wolbachia* only around 5% died (figure 1*a,b*), thus confirming the protective effect of *Wolbachia*. Moreover, the frequency of the *pastrel* resistant allele was significantly higher in flies surviving the virus infection in the absence of *Wolbachia*, whereas no significant change was detected in the presence of *Wolbachia* (figure 1*c,d*). Therefore, the benefit of the resistant allele of *pastrel* to DCV-infected flies is weaker in the presence of *Wolbachia*.

### (b) Artificial selection increases *Drosophila* C virus resistance

**Figure 2.**
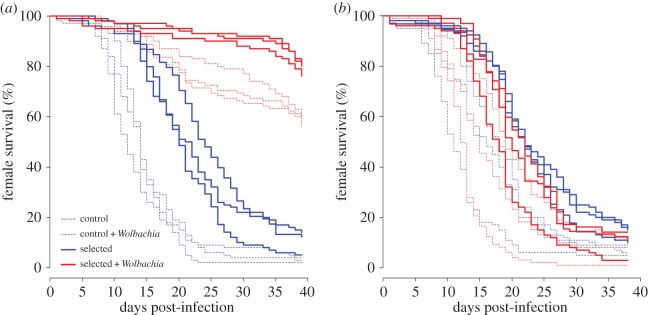
Survival of female flies upon DCV infection after selection. (*a*) Susceptibility to DCV at the end of the selection experiment and (*b*) after subsequent *Wolbachia* removal. Curves show for each replicate population the average proportion of live flies after infection.

Over nine generations we infected *Wolbachia-*infected and *Wolbachia-*free populations of *D. melanogaster* with DCV, and then measured whether resistance to the virus had increased. Upon DCV infection, the survival of the selected populations had increased relative to the controls, regardless of whether they were infected with *Wolbachia* (figure 2*a* and table 2*a*; Tukey HSD, both *p* < 0.0001). As expected, the populations that were infected with *Wolbachia* also had substantially higher survival rates (figure 2*a* and table 2*a*; Tukey HSD, both *p* < 0.0001). To check whether the change in survival reflected a change in DCV resistance, we also mock-infected flies with saline solution. These control flies all showed high survival, and there was no effect of the selection treatment or *Wolbachia* on their mortality rate (electronic supplementary material, figure S1*a*; table 2*b*).

**Table 2.** Significance of fixed effects in Cox's mixed-effect models of fly survival. In each model, the replicate populations and the vials within populations were treated as random effects.

	tetracycline-treated after selection	infection treatment	fixed effects	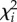	d.f.	*p*-value
(A)	no	DCV-infected	selection for DCV resistance	29.01	1	<0.001
			presence/absence of *Wolbachia*	47.75	1	<0.001
			selection-by-*Wolbachia* interaction	0.43	1	0.51
(B)	no	mock-infected	selection for DCV resistance	2.92	1	0.09
			presence/absence of *Wolbachia*	1.69	1	0.19
			selection-by-*Wolbachia* interaction	0.69	1	0.41
(C)	yes	DCV-infected	selection for DCV resistance	6.99	1	0.01
			presence/absence of *Wolbachia*	0.45	1	0.50
			selection-by-*Wolbachia* interaction	0.36	1	0.55
(D)	yes	mock-infected	selection for DCV resistance	0.18	1	0.66
			presence/absence of *Wolbachia*	0.21	1	0.65
			selection-by-*Wolbachia* interaction	2.26	1	0.13

### (c) Host-resistant allele reaches a lower frequency in populations infected with *Wolbachia*

**Figure 3.**
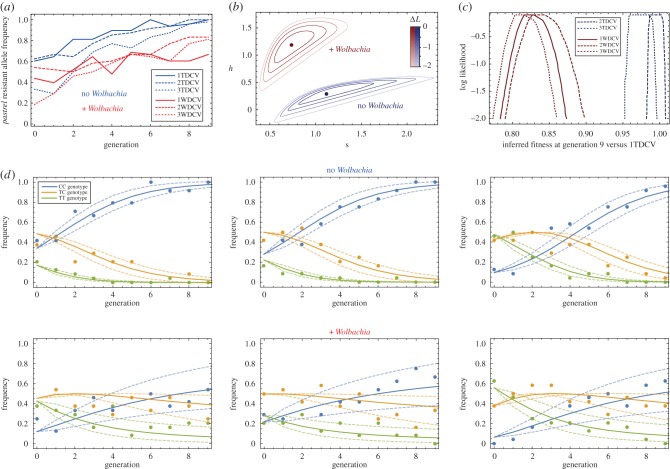
Effect of *Wolbachia* on selection acting on the resistant allele of *pastrel* in populations exposed to DCV. (*a*) Observed frequency of the *pastrel* resistant allele across generations. Each curve stands for a replicate population. (*b*) Inferred selection and dominance coefficients acting on *pastrel*. The blue and red dots represent the optimal log likelihood for the selected *Wolbachia*-free and *Wolbachia*-infected treatments, respectively. Surrounding lines show approximate contours of each likelihood surface. (*c*) Likelihood surfaces showing the relative fitnesses of the evolved populations, upon removal of *Wolbachia*. Fitness values are normalized such that the population 1TDCV has fitness equal to 1. (*d*) Change in the frequencies of *pastrel* genotypes across generations for each replicate population exposed to DCV. Blue, resistant homozygotes (CC); orange, heterozygotes (CT); green, susceptible homozygotes (TT). Dots indicate observed frequencies. Solid lines show the mean frequencies estimated from the selection model and dotted lines an interval of two standard deviations from the mean.

To investigate how *Wolbachia* affected the strength of selection on *pastrel*, we followed the frequency of the *pastrel* resistant allele across the nine generations of selection. The resistant allele was initially at intermediate frequencies and increased in frequency across generations in all DCV-exposed populations (electronic supplementary material, table S1*a*; figure 3*a*). However, the rate of increase was slower in the *Wolbachia*-infected populations (electronic supplementary material, table S1*a*; figure 3*a*). In the absence of *Wolbachia* the resistant allele was fixed, but it only reached a mean frequency of 77% in the *Wolbachia*-infected populations. In control populations that were not exposed to DCV there was a slight overall decrease in *pastrel* resistant allele frequency between the beginning and the end of the selection experiment (electronic supplementary material, table S1*b* and figure S2*a*) but no effect of *Wolbachia* (electronic supplementary material, table S1*b*).

To quantify the effect of *Wolbachia* on the strength of selection*,* we estimated the selection coefficient *s* and the dominance coefficient *h* of the *pastrel* resistant allele*.* We modelled the average fitness of the three genotypes 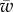 as:

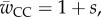


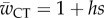


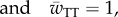
where T is the susceptible allele and C the resistant allele.

Using this model to estimate changes in genotype frequency during selection, there is a striking effect of *Wolbachia.* In populations with the symbiont, 50% or less of the population is homozygous for the resistant allele (figure 3*d*). However, in the symbiont-free populations approximately 90% or more of the populations are homozygous resistant (figure 3*d*). Plotting the likelihood surface for our estimates of *s* and *h* from the model clearly highlighted a difference in the mode of selection between the two populations (figure 3*b*).

Results from the evolutionary model showed that, in the absence of *Wolbachia*, the homozygote resistant genotype was clearly fitter than the heterozygote or homozygote susceptible genotypes (figure 3*b*). However, in the presence of *Wolbachia*, the maximum-likelihood fitness of the heterozygote genotype was increased (relative to the fitness of the susceptible genotype in the same environment), while the fitness of the homozygote-resistant genotype was decreased, such that the relative ordering of these fitnesses could not be firmly established. As a consequence, and in agreement with the observed data, the heterozygote genotype was inferred to exist in the population at significant frequencies in *Wolbachia*-infected populations at the end of the experiment (figure 3*d*).

In the control populations that were not infected with DCV there was no evidence of selection favouring either the resistant or susceptible allele of *pastrel.* The, resistant homozygotes, heterozygotes and susceptible homozygotes were all inferred to have similar fitnesses in both the *Wolbachia*-free and *Wolbachia*-infected populations (electronic supplementary material, figure S2*b,c*).

### (d) Changes in allele frequency correlate with *Drosophila* C virus resistance

The inferred evolutionary model suggests that flies evolved in the presence of *Wolbachia* would have a reduced inherent viral resistance, when *Wolbachia* was removed, than those flies that had been selected for without symbiont protection. The mean fitnesses of fly populations evolved with *Wolbachia* were inferred to be between 75% and 90% of the equivalent values for fly populations that had evolved without symbiont protection (figure 3*c*). To examine this experimentally, we treated all populations with tetracycline for two generations and examined the resistance to DCV after the removal of *Wolbachia*. Populations that had been selected for DCV resistance survived longer (figure 2*b* and table 2*c*). Although populations selected in the presence of *Wolbachia* tended to be more susceptible than those selected without the symbiont (figure 2*b*), this difference was not statistically significant (table 2*c*). This might be the result of a lack of statistical power due to the strong between-replicate variation, especially in the control populations. Alternatively, there could be other explanations such as the involvement of polymorphisms other than *pastrel* or the presence of transgenerational effects affecting DCV resistance. Mock-infected flies all showed high survival, and there was no effect of the selection treatment or *Wolbachia* (electronic supplementary material, figure S1*b*; table 2*d*).

**Figure 4.**
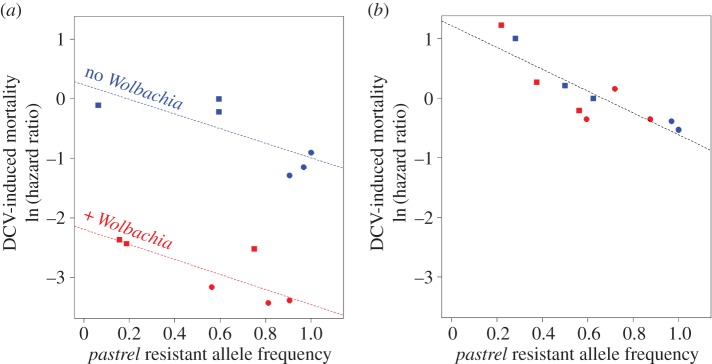
Correlation between DCV-induced mortality and the frequency of the *pastrel* resistant allele. Each dot represents the mean value of the trait for a given population that evolved without (blue) or with *Wolbachia* (red), (*a*) at the end of the selection experiment and (*b*) after subsequent *Wolbachia* removal. Squares: control populations; circles: selected populations. DCV-induced mortality is expressed as the ln of the hazard ratio estimated using a Cox's mixed-effect model. The hazard ratio is the probability of mortality at a given time point relative to a control treatment. Here the control is the replicate population 1TC that belongs to the control for the selection treatment (not selected for DCV resistance). Dashed lines indicate regressions inferred from a linear model.

As the presence of *Wolbachia* was associated with a lower frequency of the resistant allele of *pastrel*, we examined how the frequency of the allele correlated with changes in resistance. To do this, we compared the survival rates and allele frequency estimates described above. Before the populations were cured of *Wolbachia*, the frequency of *pastrel* resistant allele was negatively correlated with the DCV-induced mortality (linear model: *F*_1,8_ = 16.87; *p* = 0.003; figure 4*a*). *Wolbachia* greatly increased resistance also (linear model: *F*_1,8_ = 136.2; *p* < 0.0001; figure 4*a*), but there was no interaction between the effects of the symbiont and *pastrel* (linear model: *F*_*1*,*8*_ = 0.05; *p* = 0.83; figure 4*a*). The presence of *Wolbachia* can explain 85% of the variation in resistance among populations, while *pastrel* frequency explains only 10%. After removal of *Wolbachia,* the frequency of the *pastrel* resistant allele was also negatively correlated with virus-induced mortality (*r* = −0.86; d.f. = 10; *p* = 0.0003) and can explain 77% of the variation in resistance (figure 4*b*). Therefore, the frequency of the resistant allele of *pastrel* in a population affects its resistance to DCV.

### (e) Selection for *Drosophila* C virus resistance did not affect *Wolbachia* density

As higher densities of *Wolbachia* are associated with higher protection against viruses, we tested whether we selected for higher symbiont densities in the populations exposed to DCV. We found no evidence that this had occurred, as selected and control populations had similar symbiont densities (linear mixed-effect model: d.f. = 1; *p* = 0.69; electronic supplementary material, figure S3).

## 4. Discussion

We have found that the presence of a protective symbiont in a population can affect how selection acts on host alleles that protect against infection. We, therefore, suggest that one long-term consequence of being associated with a defensive symbiont could be that conventional immune defences encoded by the host genome become less effective in individuals without the symbiont, such that losing the symbiont would leave the host population vulnerable to infection. This may result in the host population becoming dependent on its symbiont to ensure resistance against natural enemies—a form of evolutionary ‘addiction’ where the symbiont substitutes for host immune defences. If the selection exerted by pathogens is durable, then symbiont infection could become a state from which a host population cannot escape.

We investigated the interaction between *D. melanogaster* and its viral pathogen DCV, where the main factors that determine host susceptibility are the presence of the symbiont *Wolbachia* [4,8,20,21] and a polymorphism in the host-gene *pastrel* [27,28]. In populations where all the individuals were infected with *Wolbachia*, we found that exposure to DCV led to the resistant allele of *pastrel* reaching a lower frequency than in symbiont-free populations. The presence of *Wolbachia* substantially altered the relative fitnesses of both the homozygote- and heterozygote-resistant genotypes, suggesting that the symbiont may alter the fitness landscape of host resistance in complex ways. It is conceivable that the DCV-induced mortality may follow a nonlinear relationship with the amount of virus within the flies so that the lower virus titres reached in the presence of *Wolbachia* could blur the difference in fitness between heterozygotes and resistant homozygotes. Removing the symbiont alters the fitness landscape experienced by the host, reducing the fitness of virus-infected hosts compared with populations that evolved without the symbiont. While noting a clear difference between the observed populations, we note that the estimated selection and dominance coefficients should be treated with some caution as they may be affected by unknown complexities that are not accounted for in our model. For example, there may be multiple alleles of *pastrel* [27], infection itself may have transgenerational effects on resistance, or other loci may modify the effect of *pastrel*.

*Wolbachia* is thought to infect 52% of terrestrial arthropod species [33], and in the laboratory as many as half of the strains sampled confer resistance to viruses in a *Drosophila* host [21]. Although it is not clear yet the extent to which *Wolbachia*-mediated protection is at play in natural conditions, it clearly has the potential to have an important influence on the evolution of host-encoded antiviral resistance in many species. Antiviral immune genes would be a good model to test such a hypothesis as they often evolve exceptionally fast, which is thought to be due to an arms race with viruses [34–36]. We would predict that insect taxa in which *Wolbachia* is highly prevalent may show slower rates of evolution of these genes.

A key feature of our experimental design is that all individuals in the symbiont-infected populations carried *Wolbachia*, which reflects many natural populations where *Wolbachia* is near fixation. This is often thought to be because the symbiont is causing cytoplasmic incompatibility [24], and the prevalence of the symbiont is, therefore, independent of its defensive role. While this situation may be common for *Wolbachia,* other defensive symbionts are present at an intermediate prevalence in the population [37–39]. Here the dynamics of host resistance alleles and defensive symbionts may be more complex, as changes in host resistance may alter symbiont prevalence and vice versa. We would, therefore, caution that care should be taken before extrapolating our findings to all defensive symbionts.

Several other factors may play a key role in determining whether hosts rely on defensive symbionts or their own immune defences. One of the most important is the level of resistance provided by symbionts relative to nuclear genes, as well as the range of pathogens that they provide protection against. Both *pastrel* and *Wolbachia* have substantial effects on DCV resistance. However, *Wolbachia* protects against a broad-range of RNA viruses [4,9,21,40], whereas *pastrel* and other genetic polymorphisms in *D. melanogaster* are much more specific [27,28]. This could favour the defensive symbiont over nuclear-based defences, especially if there is little genetic resistance to some viruses.

The second key factor that may differ between the two types of defence is the cost of carrying resistance genes compared with defensive symbionts. High levels of virus resistance require *Wolbachia* to be at a high density within-host tissues [20,21,41], and this correlates with reductions in survival and fecundity [15,17,18]. The costs of host-resistance genes in *Drosophila* are thought to be low. For instance, populations selected for pathogen resistance, including resistance to DCV do not exhibit decreased fitness, even under stressful conditions [42]. We also found that, in the absence of virus, the predicted fitnesses of *pastrel*-resistant homozygotes and heterozygotes are similar to the fitness of susceptible homozygotes, suggesting the absence of strong costs associated with the *pastrel* resistant allele. Overall, it seems likely that symbiont-mediated protection is a more costly form of defence in this system.

Finally, a number of other factors may tip the balance in the favour of defensive symbionts or host genes. If symbiont transmission between generations is imperfect, then the symbiont will spread more slowly. However, symbiont protection can spread in a population even if pathogens are rare if the symbiont is also able to manipulate its host reproduction [43]. Although *Wolbachia* shows a rather poor ability to manipulate reproduction in *D. melanogaster* [44,45], in other host species it induces strong sex-ratio distortion or cytoplasmic incompatibility that drives it through host populations independently of any beneficial effects [24,46].

Pathogens will also select for host and symbiont genes that increase the level of protection provided by the symbiont. In our experiments, this could be achieved by increasing the within-host density of *Wolbachia*, as antiviral protection is tightly linked to symbiont density and *Wolbachia* strains within *D. melanogaster* populations vary genetically in their density [19]. However, we did not observe such a change, suggesting that there was insufficient time, genetic variation or selection for this to occur. In particular, the symbiont strains that reach the highest density can reduce the lifespan of flies or other fitness-related traits [15,17–19], and this may have prevented them from spreading in the population.

Defensive symbionts have been described in several associations, but their impact on the evolution of host defences has been poorly explored (but see [23]). We have shown that such symbionts have the potential to influence the short-term and possibly the long-term evolution of insect defences against viruses. Investigating how insect populations respond to the presence of symbionts is a prerequisite to understand the evolution of symbioses. From an applied perspective, it is becoming more important to predict host evolutionary responses to the presence of defensive symbionts, as *Wolbachia* is being introduced on a large scale into mosquito populations to block the transmission of arboviruses [47,48].

## Supplementary Material

Supplementary material S1

## Supplementary Material

Supplementary material S2

## Supplementary Material

Table S1. Significance of fixed effects in Generalized linear mixed-effects models of pastrel resistant allele frequency.

## Data accessibility

Data are available at Dryad: http://dx.doi.org/10.5061/dryad.2v0m8.
