# Supplementary material for: Addicted? Reduced host resistance in populations with defensive symbionts
Source: Proc Biol Sci. 2016 Jun 29;283(1833):20160778. doi: 10.1098/rspb.2016.0778 (PMC4936038; doi:10.1098/rspb.2016.0778)
Supplement: Supplementary material S1 [file rspb20160778supp1.docx]

**Supplementary material S1**

**Virus production**

DCV was produced in Schneider Drosophila line 2 (DL2) cells as described in [1]. Cells were cultured at 26.5°C in Schneider’s Drosophila Medium with 10% Fetal Bovine Serum, 100 U/ml penicillin and 100 mg/ml streptomycin (all Invitrogen, UK). Cells were then freeze-thawed twice to lyse cells and centrifuged at 4000 g for 10 minutes at 4°C to remove cellular debris. Virus was then aliquoted and frozen at -80°C. Virus infectivity, was calculated using serial dilutions of virus in Schneider’s medium added to wells of a plate of DL2 cells as described in [1]. After 7 days the wells were visually examined under the microscope and classed as “infected” when cell death (presence of cell debris) and cytopathic effects were visible (lysing, shrinking or losing of compartmentation of cells). The Tissue Culture Infective Dose 50 (TCID50) was calculated by the Reed-Muench end-point method [2].

**DNA extraction**

For genotyping, DNA from individual female flies was extracted by crushing the fly in 150 µL of a 5% w/v suspension of Chelex 100 resin (Sigma-Aldrich) and 1 µL of proteinase K (20 mg⁄mL, Fermentas). Extracts were incubated for five hours at 56°C. After 10 min at 95°C, samples were centrifuged and stored at -20°C.

To measure the *Wolbachia* density within fly tissues after the selection experiment, DNA from a pool of ten 3-6 day-old females was extracted using the Gentra Puregene kit (Qiagen). Ten pools of females were extracted for each *Wolbachia*-infected replicate population.

**Genotyping**

The *pastrel* genotype of female flies collected during the course of the selection experiment was determined using quantitative PCR (qPCR). We also genotyped males from generation 0 and generation 9 in the DCV-exposed populations with and without *Wolbachia*. We found that the frequency of the *pastrel* resistant allele was strongly correlated between males and females (r = 0.96; P < 0.0001), therefore we decided to focus on females only for the rest of the genotyping. Two forward allele-specific primers were designed, with the last base corresponding to the allele on the SNP and the penultimate base differing from the target sequence to increase the qPCR specificity (5’-GCATGGTGTCCATGAAGAC-3’ for allele C which is associated with resistance and 5’-GCATGGTGTCCATGAAGAT-3’ for allele T which is associated with susceptibility). For each individual, one qPCR reaction per allele was performed using the corresponding forward primer and the reverse primer 5’-TCCTCGACAGGAACCCAGTA-3’, amplifying a 230bp fragment. The qPCR cycle was 95°C for 2 min, followed by 40 cycles of 95°C for 5 s, 60°C for 30 s. The accuracy of the genotyping by qPCR was checked using five isogenic fly lines of the DGRP panel [3] for which the genome sequence is known (Lines 26, 38, 85, 228, 101). Different combinations of F1 hybrids between these lines were also tested as controls for heterozygote genotypes.

The individual critical thresholds (*Ct*) obtained from the qPCR were corrected for variation in qPCR efficiency between the two alleles by subtracting the mean *Ct* of positive DGRP controls averaged over several qPCR plates. To determine whether a given allele was present or absent, we calculated for a given allele the difference between its *Ct* and the *Ct* of the alternative allele. Based on the DGRP control lines for which the genotype is known, we estimated that a difference of less than 2 between *Ct* values allows us to call the right allele when present and avoids calling false-positive. The accuracy of this method was checked by Sanger sequencing 29 individuals from the experimental populations. To do this, we used primers amplifying a 328bp region spanning the SNP of interest (5’-CGTCCTCGAAGTTCCGTAAA-3’ and 5’-GCGAGCTAGTGTACAACATAACC-3’). We found that chromatograms from the Sanger sequencing either showed one of the two *pastrel* C521T alleles, indicating homozygote individuals, or an ambigous base with clear signals for both C521T alleles, indicating heterozygotes. Our genotypes based on the qPCR were all consistent with the Sanger data.

In total, 1,440 and 672 females were genotyped for the DCV-selected populations (24 females per replicate population per generation) and for control populations (24 females per replicate population at generation 0 and 9 ; 8 females per population for the rest) respectively.

***Wolbachia* PCR screening and *Wolbachia* density**

The *Wolbachia* infection status of the replicate populations was checked by PCR one generation before the selection experiment on 30 individual females using primers wsp81F and wsp691R as described in [4].

In order to measure *Wolbachia* density, the copy number of the *Wolbachia* gene *atpD* (atpDQALL_F: 5’-CCTTATCTTAAAGGAGGAAA-3’; atpDQALL_R: 5’- AATCCTTTATGAGCTTTTGC-3’) relative to the endogenous control gene *actin 5C* (Forward primer: 5’-GACGAAGAAGTTGCTGCTCTGGTTG-3’; Reverse primer: 5’-TGAGGATAC CACGCTTGCTCTGC-3’) was quantified using the SensiFAST SYBR & Fluorescein kit (Bioline). The *Wolbachia* density was estimated as: *2^ΔCt^*, where *Ct* is the mean cycle threshold of two technical replicates and *ΔCt=Ct_actin5C_-Ct_atpD_*. The qPCR cycle was 95°C for 2 min, followed by 40 cycles of 95°C for 5 s, 60°C for 30 s.

1. Longdon, B., Cao, C., Martinez, J. & Jiggins, F. M. 2013 Previous exposure to an RNA virus does not protect against subsequent infection in Drosophila melanogaster. *PLoS One* **8**, e73833. (doi:10.1371/journal.pone.0073833)

2. Reed, L. & Muench, H. 1938 A simple method of estimating fifty per cent endpoints. *Am. J. Hyg.* **27**, 493–497.

3. Mackay, T. F. C. et al. 2012 The Drosophila melanogaster Genetic Reference Panel. *Nature* **482**, 173–178. (doi:10.1038/nature10811)

4. Braig, H. R., Zhou, W., Dobson, S. L. & O’Neill, S. L. 1998 Cloning and characterization of a gene encoding the major surface protein of the bacterial endosymbiont Wolbachia pipientis. *J. Bacteriol.* **180**, 2373–2378. (doi:10.1099/0022-1317-69-1-35)
