# Supplementary material for: Addicted? Reduced host resistance in populations with defensive symbionts
Source: Proc Biol Sci. 2016 Jun 29;283(1833):20160778. doi: 10.1098/rspb.2016.0778 (PMC4936038; doi:10.1098/rspb.2016.0778)
Supplement: Supplementary material S2 [file rspb20160778supp2.docx]

**Supplementary material S2**


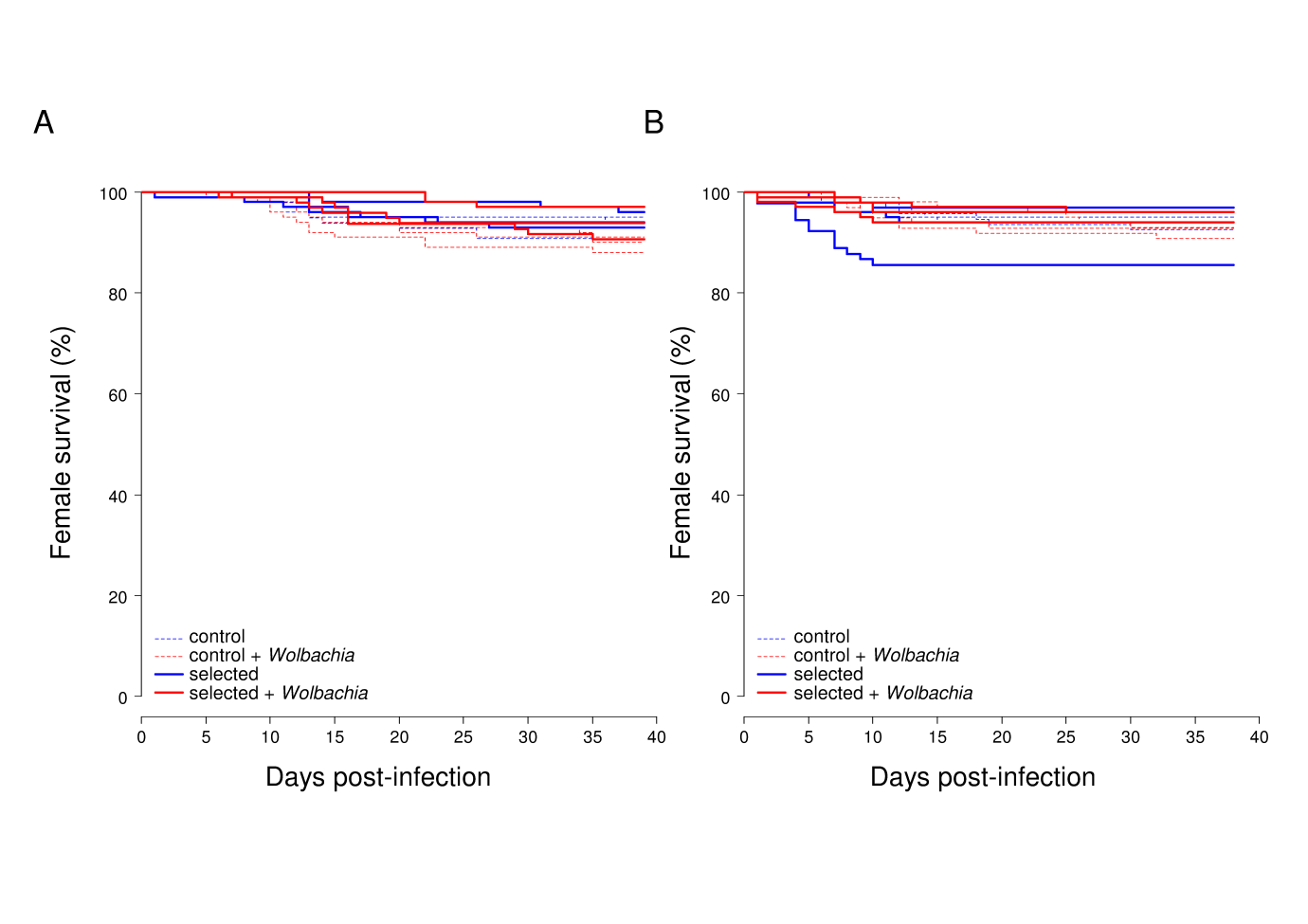


**Figure S1. Survival of female flies upon infection with Ringer’s solution.**

(A) Susceptibility to mock infection at the end of the selection experiment and (B) after subsequent *Wolbachia*-removal. Curves show for each replicate population the average proportion of live flies after infection.


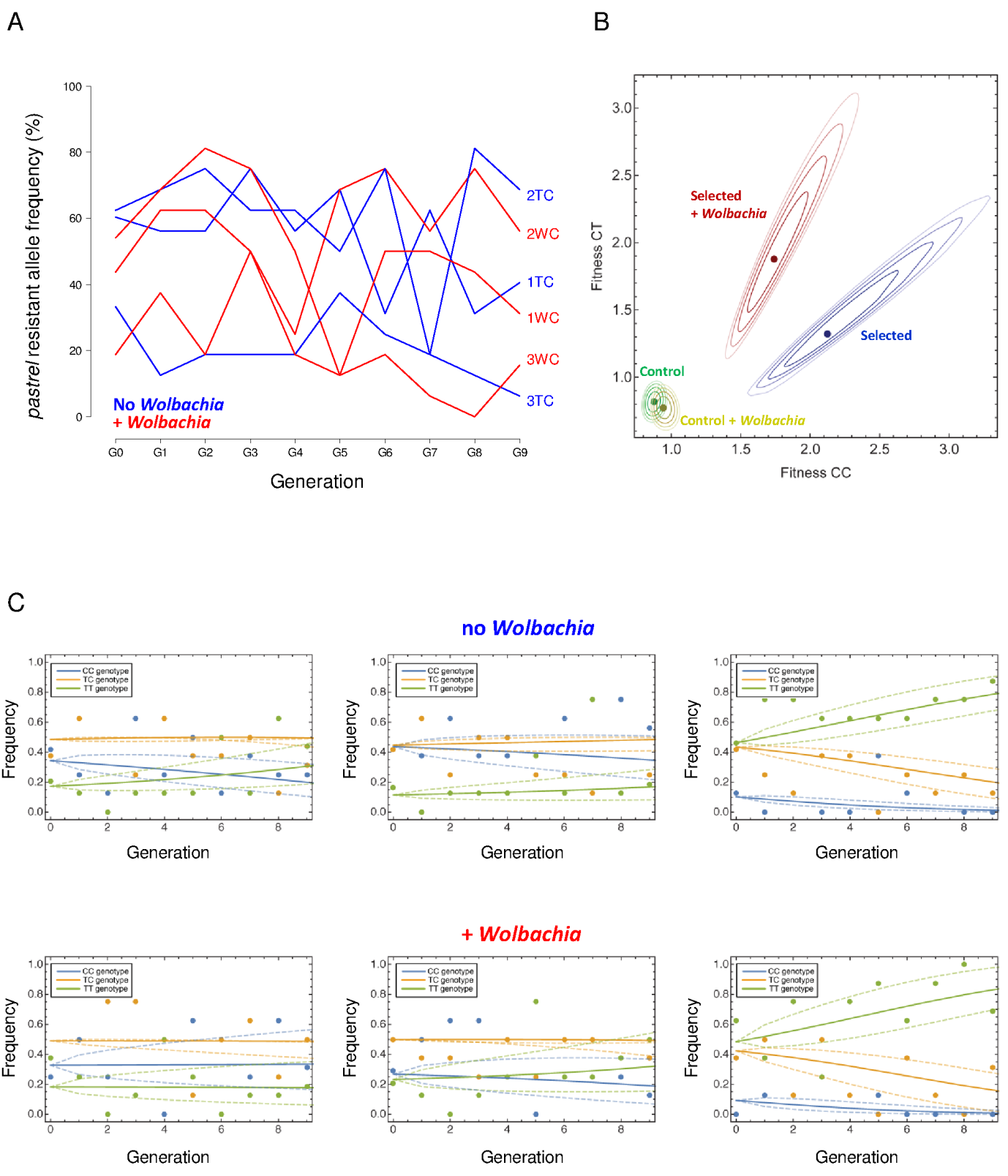


**Figure S2. Effect of *Wolbachia* on selection acting on the resistant allele of *pastrel* in control populations that were not exposed to DCV.**

(A) Observed frequency of *pastrel* resistant allele across generations in control populations. Each curve stands for a replicate population with blue and red curves representing *Wolbachia*-cured and *Wolbachia*-infected populations respectively. (B) Inferred fitnesses of pastrel resistant homozygotes CC and heterozygotes CT relative to the fitness of susceptible homozygotes TT.  Dots show the mean relative fitnesses and surrounding lines show approximate contours of each likelihood surface. (C) Change in the frequencies of *pastrel* genotypes across generations for each replicate control population. Blue: resistant homozygotes (CC); orange: heterozygotes (CT); green: susceptible homozygotes (TT). Dots indicate observed frequencies. Solid lines show the mean frequencies estimated from the selection model and dotted lines an interval of two standard deviations from the mean.


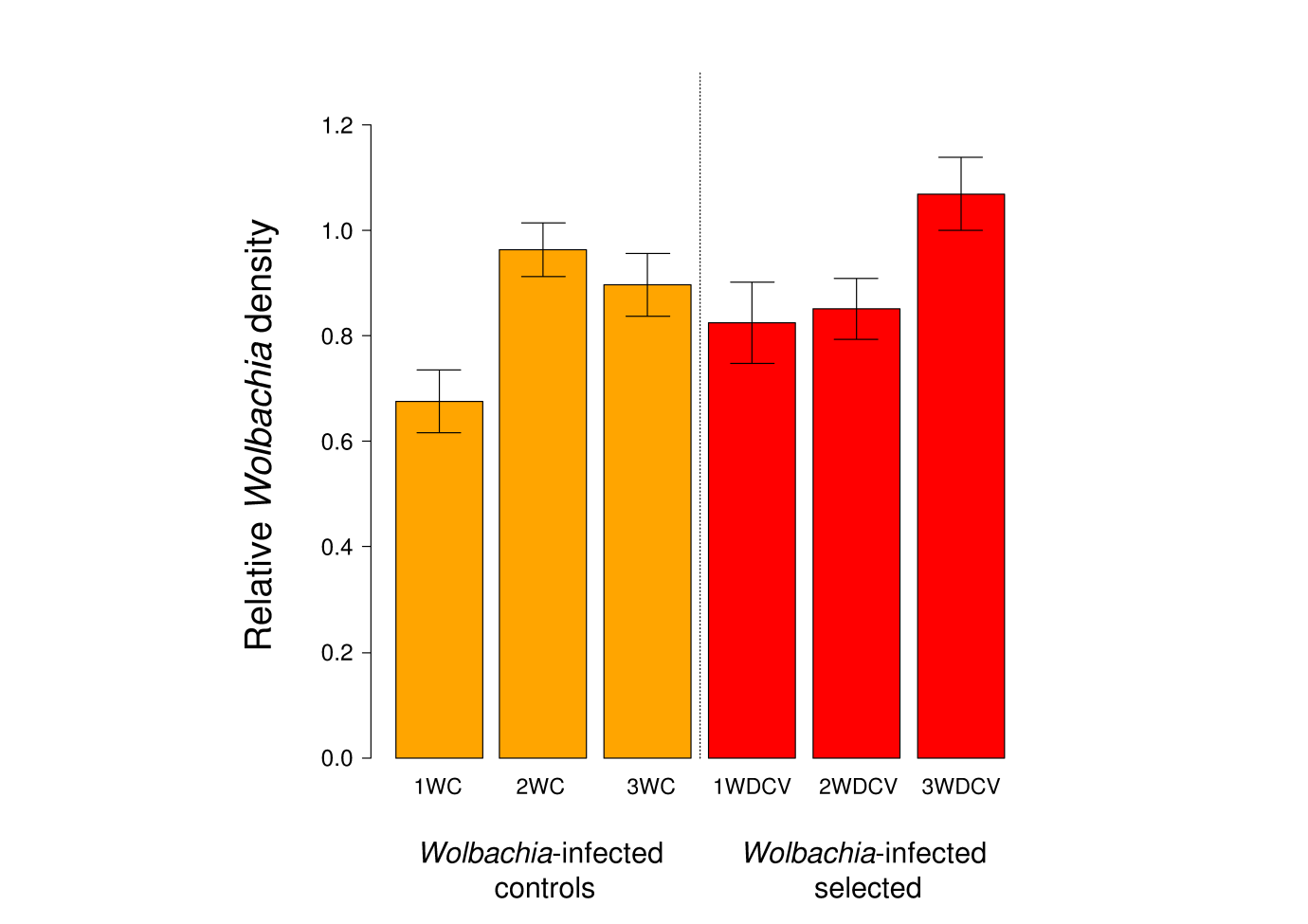


**Figure S3. *Wolbachia* density at the end of the selection experiment.**

*Wolbachia* density is expressed as the ratio of the *Wolbachia* gene *atpD* to the *Drosophila* gene *actin5C*. Orange: *Wolbachia*-infected control populations; red: *Wolbachia*-infected selected populations. Each bar represents the mean value of the trait for a given replicate population. Error bars are standard errors.
