# Supplementary material for: Addicted? Reduced host resistance in populations with defensive symbionts
Source: Proc Biol Sci. 2016 Jun 29;283(1833):20160778. doi: 10.1098/rspb.2016.0778 (PMC4936038; doi:10.1098/rspb.2016.0778)
Supplement: Table S1. Significance of fixed effects in Generalized linear mixed-effects models of pastrel resistant allele frequency. [file rspb20160778supp3.docx]

**Table S1. Significance of fixed effects in Generalized linear mixed-effects models of *pastrel* resistant allele frequency.**

In each model, the replicate population was treated as a random effect.

|  | Selection treatment | Fixed effects | χ_i_² | d.f. | *P* |
| --- | --- | --- | --- | --- | --- |
| (A) | DCV | Generation | 339.8 | 2 | < 0.0001 |
|  |  | *Wolbachia* | 53.08 | 2 | < 0.0001 |
|  |  | Generation-by-*Wolbachia* interaction | 45.35 | 1 | < 0.0001 |
|  |  |  |  |  |  |
| (B) | Control | Generation | 10.03 | 1 | 0.002 |
|  |  | *Wolbachia* | 0.09 | 1 | 0.76 |
|  |  | Generation-by-*Wolbachia* interaction | 0.41 | 1 | 0.52 |
